# Effect of cocoa powder on hypertension and antioxidant status in uninephrectomized hypertensive rats

**DOI:** 10.14202/vetworld.2020.695-705

**Published:** 2020-04-16

**Authors:** Olayinka Christianah Jayeola, Ademola Adetokunbo Oyagbemi, Omolara Ibiwunmi Okunlola, Olayiwola Olubamiwa, Temidayo Olutayo Omobowale, Temitayo Olabisi Ajibade, Foluso Bolawaye Bolaji-Alabi, Blessing Seun Ogunpolu, Olufunke Olubunmi Falayi, Adebowale Benard Saba, Adeolu Alex Adedapo, Momoh Audu Yakubu, Afolabi Oluwadun, Oluwafemi Omoniyi Oguntibeju

**Affiliations:** 1 Cocoa Research Institute of Nigeria, P.M.B 5244, Ibadan, Oyo State, Nigeria; 2 Department of Veterinary Physiology and Biochemistry, Faculty of Veterinary Medicine, University of Ibadan, Ibadan, Nigeria; 3 Standard Organization of Nigeria, Block 7, Obafemi Awolowo Way, Alausa Ikeja, Lagos, Nigeria; 4 Department of Veterinary Medicine, Faculty of Veterinary Medicine, University of Ibadan, Nigeria; 5 Department of Veterinary Theriogenology, Faculty of Veterinary Medicine, University of Ibadan, Ibadan, Nigeria; 6 Department of Veterinary Pharmacology and Toxicology, Faculty of Veterinary Medicine, University of Ibadan, Ibadan, Nigeria; 7 Department of Environmental and Interdisciplinary Sciences, College of Science, Engineering and Technology, Vascular Biology Unit, Center for Cardiovascular Diseases, College of Pharmacy and Health Sciences (COPHS), Texas Southern University, Houston, Texas, USA; 8 Department of Medical Microbiology, Olabisi Onabanjo University, Sagamu Campus, Sagamu, Nigeria; 9 Phytomedicine and Phytochemistry Group, Department of Biomedical Sciences, Faculty of Health and Wellness Sciences, Cape Peninsula University of Technology, Bellville 7535, South Africa

**Keywords:** antioxidant therapy, cocoa powder, high salt diet, hypertension, oxidative stress

## Abstract

**Background and Aim:**

High salt diet and uninephrectomy are associated with high blood pressure with attendant cardiovascular disease conditions such as hypertension, renal damage, myocardial infarction, and stroke. The aim of this study was to investigate the beneficial effects of consumption of cocoa and cocoa-containing products in the management of high blood pressure in uninephrectomized hypertensive rats.

**Materials and Methods:**

The effect of cocoa powder on blood pressure, markers of inflammation, oxidative stress, and histopathology were investigated in uninephrectomized animals fed with cocoa feed alone or in combination with a high salt diet. Male rats were randomly divided into five groups: Group A was the control group and fed with normal feed alone, Group B was fed with cocoa feed alone, Group C was fed with high salt diet (8% salt), GroupD was fed with cocoa-feed compounded with 8% salt for 4weeks after uninephrectomy, and GroupE was uninephrectomized rats on a normal diet. The left kidneys of animals in GroupsC, D, and E were removed by surgery. After 4weeks of treatment, the systolic, diastolic, and mean arterial blood pressure was measured. The serum markers of renal damage and oxidative stress were determined. Histological examination was also performed on renal and cardiac tissues.

**Results:**

Results showed significant increases in biomarkers of oxidative stress, inflammation, and renal damage with a concomitant decrease in antioxidant status in hypertensive uninephrectomized rats. Cocoa feed, however, significantly improved blood pressure and nitric oxide bioavailability, antioxidant status and reduced markers of inflammation and oxidative stress.

**Conclusion:**

These findings show that cocoa powder could be used to maintain blood pressure levels in hypertensive rats through its antioxidant capacity.

## Introduction

The kidneys are very important organs that are involved in the maintenance of arterial blood pressure and modulation of hypertension. While uninephrectomy has been said to have less impact on human well-being, however, studies have also shown that it protects a subsequent increase in blood pressure[1]. However, a compensatory mechanism is developed in uninephrectomized animals which allow the animal to maintain normal blood pressure, in complicated events such as in high salt diet, a uninephrectomized animal develops high blood pressure in addition to increased diuresis[2]. Furthermore, compared to medieval times, modern human consumption of salt has greatly increased, and much evidence has proven salt to be a major risk factor in various cardiovascular and renal diseases. Salt overload can precipitate inhibition of sodium pump, with resultant elevation of intracellular sodium, causing influx of calcium ions into cells, increase contraction and peripheral resistance, and triggering hypertension [3]. Cocoa butter contains significant amounts of fatty acids, whereas the nonfat cocoa solids contain vitamins, minerals, fiber, and polyphenols [4]. The polyphenols-rich cocoa products have been shown to diminish obesity-mediated metabolic diseases through the inhibition of chronic inflammation [5,6]. Cocoa has been reported to increase postprandial high-density lipoprotein-cholesterol in diabetic individuals [7]. The antioxidant, anti-inflammatory, and free-radical scavenging activities of cocoa powder have also been documented [8,9]. Similarly, cocoa as the diet has been documented to have a prophylactic effect against malaria[10]. Cocoa and chocolate products have been reported to maintain blood pressure and prevent endothelial dysfunction together with antidiabetic properties [11-13].

It should be noted that although sodium is an important nutrient in the maintenance of good health as it is required for nerve conduction, cell signaling, maintenance of plasma volume, and some other biochemical processes [14]. Excess sodium intake is associated with an increase in blood pressure levels and subsequent risk of cardiovascular and renal diseases. Different approaches have been taken to control sodium consumption, including regulating salt content in commercial foods, establishing policy goals, education and extension services, and sanctioning defaulters[15]. Adverse effects associated with uninephrectomy are usually due to a decrease in nephron number, and it has been shown that loss of nephrons is related to harmful renal outcomes [16]. Usually, following uninephrectomy, the remaining kidney undergoes a compensatory enlargement, triggered by increased blood flow and increased transport of amino acids. This compensatory enlargement, however, does not compensate for the metabolic functions needed [17]. The impaired ability of the kidneys of hypertensive rats to excrete sodium in response to increase in blood pressure levels might be worsened with uninephrectomy.

This study aimed to ascertain if cocoa feed can ameliorate hypertension and oxidative stress precipitated by high salt diet alongside uninephrectomy in hypertensive rats.

## Materials and Methods

### Ethical approval

The Animal Care and Use Research Ethical Committee of the Faculty of Veterinary Medicine of Ibadan, Nigeria approved the study with ethical approval number UI-ACUREC/18/0133.

### Experimental animals and design

#### Animals

In the study, 50male Wistar albino rats weighing between 150 and 200g were used. All the animals received humane care according to the criteria outlined in the Public Health Service Policy on Humane Care and Use of Laboratory Animals [18].

#### Groups

The rats were randomly divided into five groups with ten rats (n=10) in a group: GroupA was the control group fed with normal feed alone, GroupB was fed with cocoa feed control, GroupC was fed with normal feed compounded with 8% salt, GroupD was fed with cocoa feed compounded with 8% salt, and GroupE was uninephrectomized on normal feed alone. Animals in GroupsC, D, and E underwent surgery before the start of the experiment. Experimental animals were placed on cocoa feed alone or in combination with 8% salt diet for 4weeks. The blood pressure of the rats was taken 24h after the last administration using CODA Kent scientific.

### Materials

Standardized natural cocoa powder (non-alkalized) as packaged by the Cocoa Research Institute of Nigeria (CRIN), Idi-Ayunre, Ibadan, Oyo State, Nigeria, was used for this study. Pure analytical grade sodium chloride was used for this study. Animal feed was specially formulated to consist of the normal rat diet with 2% natural cocoa powder inclusion. The feed was modified by Jayeola *et al*. [19].

#### Surgery

The rats were anesthetized using xylazine/ketamine. Briefly, the region undergoing surgery was shaved, and an incision was made on the left side. Anesthesia was induced by the intramuscular injection of ketamine and xylazine mixture [20]. The left paravertebral area was incised after sterile preparation of the area (2-3cm) with the scalpel blade to access the left kidney. The renal vessel was carefully isolated with mosquitoes’ forceps, ligated with 2-0 catgut, and the part proximal to the ligature was excised. The muscle and skin incised were later sutured after the nephrectomy with catgut (2-0) and nylon (2-0) sutures, respectively. After surgery, all animals were kept in a sterile environment to heal, with free access to the feed and water.

### Blood pressure measurements

Blood pressure parameters, including systolic blood pressure (SBP), diastolic blood pressure (DBP), and mean arterial blood pressure (MAP), were determined non-invasively in conscious animals by tail plethysmography using an automated blood pressure monitor (CODA S1, Kent Scientific Corporation, Connecticut, USA).

### Serum preparation

Approximately 3ml of blood were collected by retro-orbital venous puncture using plain capillary tubes into plain bottles and allowed to clot. The clotted blood was then centrifuged at 4000 revolutions per minute for 10min. Clear serum was separated with Pasteur pipette into another plain tube and stored at 4°C until needed.

The excised organs (kidneys and hearts) were rinsed and homogenized using 50 mM Tris-HCl buffer (pH7.4) containing 1.15% KCl. The homogenates were subjected to cold centrifugation at 4°C using a speed of 10,000*g* for 15min. The cytosolic/post-mitochondrial fractions (PMFs) obtained from cardiac and renal homogenates were used for biochemical assays.

### Biochemical analysis

#### Renal and cardiac biomarkers of oxidative stress

Hydrogen peroxide generation was determined according to the method of Wolff [21]. The reaction mixture was subsequently incubated at room temperature for 30min. The mixtures were read at 560nm, and H_2_O_2_ generated was extrapolated from the H_2_O_2_ standard curve. The malondialdehyde (MDA) content as an index of lipid peroxidation was quantified in the PMFs of cardiac and renal tissue according to the method of Varshney and Kale [22]. The absorbance was measured against a blank at 532nm. Lipid peroxidation was calculated with a molar extinction coefficient of 1.56 × 10^5^/M/cm. Protein carbonyl (PCO) contents in the renal and cardiac tissues were measured using the method of Reznick and Packer[23]. The absorbance of the sample was measured at 370nm. The carbonyl content was calculated based on the molar extinction coefficient of 2,4,-dinitrophenylhydrazine (2.210^4^ cm^1^ M^1^) and expressed as nmoles/mg protein while Vitamin C contents were measured as earlier described [24].

#### Renal and cardiac antioxidants

The superoxide dismutase (SOD) assay was carried out by the method of Misra and Fridovich with slight modification from our laboratory [25,26]. The increase in absorbance at 480nm was monitored every 30 s for 150 s. One unit of SOD activity was given as the amount of SOD necessary to cause 50% inhibition of the auto-oxidation of adrenaline to adrenochrome. The reduced glutathione (GSH) was estimated by the method of Jollow *et al*. [27]. Catalase (CAT) activity was determined according to the method of Sinha[28]. One unit of CAT activity represents the amount of enzyme required to decompose 1 µmol of H_2_O_2_/min. Glutathione peroxidase activity was also measured, according to Beutler *et al*. [29]. Glutathione S-transferase was estimated by the method of Habig *et al*. [30] using 1-chloro-2, 4-dinitrobenzene as substrate. The protein thiol (PSH) and non-protein thiol (NPSH) contents were determined, as described by Ellman [31]. Protein concentration was determined by the Biuret method of Gornal *et al*. [32] using bovine serum albumin as standard.

### Determination of serum biomarkers of renal damage and hypertension

The serum nitric oxide concentrations were measured spectrophotometrically at 548nm, according to the method of Olaleye *et al*. [33]. The serum myeloperoxidase (MPO) activity was determined according to the method of Xia and Zweier [34]. The advanced oxidation protein product (AOPP) contents were determined, as described by Kayali *et al*. [35]. Briefly, 0.4ml of cardiac and renal PMFs were treated with 0.8ml phosphate buffer (0.1 M; pH7.4). The absorbance of the reaction mixture was immediately recorded at 340nm wavelength. The content of AOPP for each sample was calculated using the extinction coefficient of 261 cm^−1^ mM^−1^ and the results were expressed as µmoles/mg protein. The activity of xanthine oxidase was determined according to the method of Akaike *et al*. [36]. The blood urea nitrogen and creatinine were determined using Randox kits following the manufacturer’s instructions.

### Histopathology

Small pieces of kidney and heart were fixed in 10% formalin, embedded in paraffin wax, and sections of 5-6mm in thickness were made and thereafter stained with hematoxylin and eosin for histopathological examination according to the methods described by Drury *et al*. [37]. The sections were examined with light microscopy.

### Statistical analysis

Data obtained were analyzed with one-way ANOVA with Dunnett’s post-test at a 95% confidence limit. All values are expressed as mean±SD. The test of significance between two groups was estimated by Student’s t-test.

## Results

### Renal and cardiac enzymic antioxidants

In this study, cardiac and renal enzymatic antioxidants such as SOD and CAT activity were significantly depleted in the 8% salt and uninephrectomy untreated, uninephrectomy alone, and the cocoa treated groups when compared with the normal control and cocoa control groups. The cocoa treated group showed increased SOD and CAT activity when compared with the untreated group (Tables-1 and 2).

**Table-1:** Effects of cocoa on cardiac enzymatic antioxidant system

Cardiac enzymatic antioxidants	Groups				

	A	B	C	D	E
SOD	4.3±0.28	4.4±0.2	2.1±0.1^a,b,d^	3.06±0.2^a,b,c^	3.02±0.6^a,b,c^
GST	4.4±1.2	5.4±2.0	18.0±1.7^a,b^	19.7±5.4^a,b^	14.2±9.2^a,b^
CAT	12.1±2.4	13.9±2.7	5.6±3.1^a,b,e^	7.7±1.4^a,b^	8.8±0.9^a,b,c^
GPx	36±3.2	37.4±3.7	36.0±3.6	39.5±5.1	37.9±5.7

Values presented as mean ± S.D. Alphabets indicate significant differences across groups at α<0.05. SOD (superoxide dismutase; units/mg protein), GST (glutathione S-transferase; mmole1-chloro-2,4-dinitrobenzene-GSH complex formed/min/mg protein), Catalase (mmole of H_2_O_2_ consumed/min/mg protein) GPx (glutathione peroxidase; units/mg protein)

A (Normal Feed alone), B (Cocoa Feed alone), C (Normal Feed + 8% salt + Uninephrectomy), D (Cocoa Feed + 8% salt + Uninephrectomy), E (Uninephrectomy alone)

**Table-2:** Effects of cocoa on renal enzymatic antioxidant system.

Renal enzymatic antioxidants	Groups				

	A	B	C	D	E
SOD	2.01±0.1	2.04±0.4	1.4±0.08^a,b,d,e^	1.74±0.14^a,b,c^	1.8±0.15^a,b,c^
GST	13.2±1.6	11.7±2.8	6.5±0.9^a,b^	6.7±1.9^a,b,e^	6.9±2.97^a,b^
CAT	2.6±1.7	0.8±0.3	0.8±0.3^a^	1.61±0.8^b,c,e^	3.5±1.1^a,b,c,d^
GPx	35.1±2.1	42.2±3.9	35.6±2.3^b^	37.0±3.4^b^	37.6±9.6

Values presented as mean ± S.D. Alphabets indicate significant differences across groups at α<0.05. SOD (superoxide dismutase; units/mg protein), GST (glutathione S-transferase; mmole1-chloro-2,4-dinitrobenzene-GSH complex formed/min/mg protein), Catalase (mmole of H_2_O_2_ consumed/min/mg protein) GPx (glutathione peroxidase; units/mg protein) A (Normal Feed alone), B (Cocoa Feed alone), C (Normal Feed + 8% salt + Uninephrectomy), D (Cocoa Feed + 8% salt + Uninephrectomy), E (Uninephrectomy alone)

### Cardiac and renal non-enzymatic antioxidants

Cardiac and renal non-enzymatic antioxidants such as reduced GSH, NPSH, PSH, and Vitamin C levels were significantly depleted in high salt diet uninephrectomized untreated, uninephrectomized alone, and in uninephrectomized cocoa treated groups when compared with the normal control. The cocoa treated and uninephrectomized alone groups exhibited a mild increase in the levels of reduced GSH, NPSH, PSH, and Vitamin C when compared with the untreated groups (Tables-3 and 4).

**Table-3:** Effects of cocoa on cardiac non-enzymatic antioxidants.

Cardiac non-enzymatic antioxidants	Groups				

	A	B	C	D	E
GSH	236.7±20.1	227.4±24	170.3±8.3	206.1±8.5	203.6±11.7
Non-Protein Thiol	40.7±2.0	42.1±7.7	29.4±0.8	33.3±1.7	34.2±3.1
Protein Thiol	34.3±2.0	32.2±1.4	33.0±0.5	32.3±3.2	33.9±7.5
Vitamin C	1.42±0.18	1.6±0.1	1.3±0.2	1.4±0.2	1.4±0.2

Values presented as mean ± S.D. Alphabets indicate significant differences across groups at α<0.05. GSH (reduced glutathione; μmol /mg protein), non-protein thiol (μmol /mg protein), protein thiol (μmol /mg protein). Vitamin C (μmol /mg protein) A (Normal Feed alone), B (Cocoa Feed alone), C (Normal Feed + 8% salt + Uninephrectomy), D (Cocoa Feed + 8% salt + Uninephrectomy), E (Uninephrectomy alone)

**Table-4:** Effects of cocoa on renal non-enzymatic anti-oxidants.

Renal non-enzymatic antioxidants	Groups				

	A	B	C	D	E
GSH	118±11	104±5.0	38.1±2.0	75.8±8.2	93.5±4.9
Non-Protein Thiol	32.9±3.0	32.7±0.12	24.5±0.5	28.3±3.2	29.1±4.4
Protein Thiol	82.6±9.7	79.2±1.3	59.9±2.6	61.7±1.9	62.8±6.1
Vitamin C	1.7±0.03	1.8±0.2	1.3±0.04	1.51±0.04	1.56±0.2

Values presented as mean ± S.D. Alphabets indicate significant differences across groups at α<0.05. GSH (reduced glutathione; μmol /mg protein), non-protein thiol (μmol /mg protein), protein thiol (μmol /mg protein). Vitamin C (μmol /mg protein) A (Normal Feed alone), B (Cocoa Feed alone), C (Normal Feed + 8% salt + Uninephrectomy), D (Cocoa Feed + 8% salt + Uninephrectomy), E (Uninephrectomy alone)

### Serum biomarkers of oxidative stress and inflammation

Serum AOPPs, MPO, and xanthine oxidase activities were significantly elevated in high salt diets and uninephrectomized untreated, and uninephrectomized treated with cocoa when compared with the normal control and the cocoa power alone. The nitric oxide levels were insignificant across the groups; the cocoa control group showed the least values of these biomarkers of oxidative stress (Table-5).

**Table-5:** Effects of cocoa on serum markers of inflammation and oxidative stress.

Serum markers of inflammation and oxidative stress	Groups				

	A	B	C	D	E
AOPP	166±1.6	145.8±8.8	200.9±4.3^a,b,d,e^	192.3±6.4^a,b,c^	191.6±7.2^a,b,c^
NO	0.9±0.04	0.85±0.06	0.87±0.02	0.75±0.18	0.8±0.13
MPO	18.4±1.8	15.2±2.4	32.3±1.6^a,b,d,e^	27.9±4.9^a,b,c^	28.3±1.8^a,b,c^
XO	1.5±0.02	1.17±0.03	3.2±0.5^a,b,d,e^	2.01±0.2^a,b,c^	1.8±0.2^a,b,c^

Values presented as mean ± S.D. Alphabets indicate significant differences across groups at α<0.05. AOPP (advanced oxidative protein product nmoles/mg protein), NO (nitric oxide; μmol/mg protein), MPO (myeloperoxidase; μmol/minute), XO (xanthine oxidase; units/min/mg protein) A (Normal Feed alone), B (Cocoa Feed alone), C (Normal Feed + 8% salt + Uninephrectomy), D (Cocoa Feed + 8% salt + Uninephrectomy), E (Uninephrectomy alone)

### Renal and cardiac markers of oxidative stress

**Figure-1:**
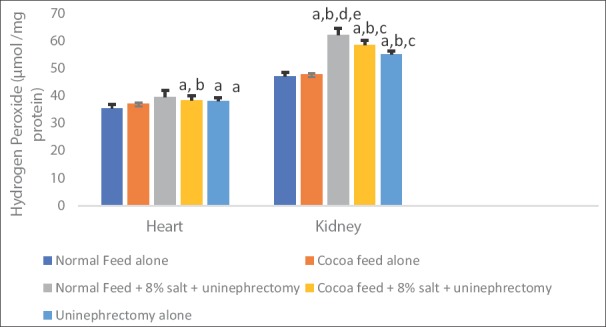
Effects of cocoa on cardiac and renal hydrogen peroxide generation. Values presented as mean ± S.D (n=10). GroupA (Normal Feed alone), GroupB (Cocoa Feed alone), GroupC Normal Feed + 8% salt + Uninephrectomy), GroupD (Cocoa Feed + 8% salt + Uninephrectomy), GroupE (Uninephrectomy alone). Alphabets indicate significant differences across groups at α<0.05. Superscript (a) indicates significant difference when compared to GroupA (Normal Feed alone), superscript (b) indicates significant difference when compared to GroupB (Cocoa Feed alone) while superscript (c) indicates significant difference when compared to GroupC Normal Feed + 8% salt + Uninephrectomy).

**Figure-2:**
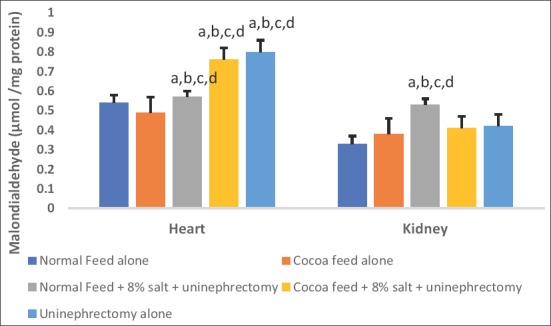
Effects of cocoa on cardiac and renal malondialdehyde (MDA) levels. Values presented as mean ± S.D (n=10). GroupA (Normal Feed alone), GroupB (Cocoa Feed alone), GroupC Normal Feed + 8% salt + Uninephrectomy), GroupD (Cocoa Feed + 8% salt + Uninephrectomy), GroupE (Uninephrectomy alone). Alphabets indicate significant differences across groups at α<0.05. Superscript (a) indicates significant difference when compared to GroupA (Normal Feed alone), superscript (b) indicates significant difference when compared to GroupB (Cocoa Feed alone) while superscript (c) indicates significant difference when compared to GroupC Normal Feed + 8% salt + Uninephrectomy).

**Figure-3:**
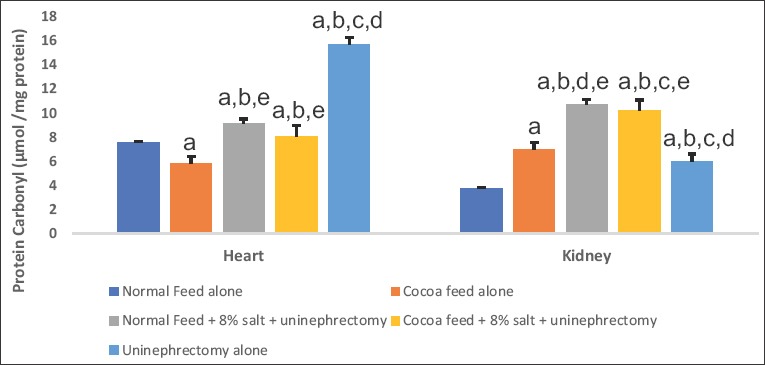
Effect of cocoa on cardiac and renal carbonyl contents. Values presented as mean ± S.D (n=10). GroupA (Normal Feed alone), GroupB (Cocoa Feed alone), GroupC Normal Feed + 8% salt + Uninephrectomy), GroupD (Cocoa Feed + 8% salt + Uninephrectomy), GroupE (Uninephrectomy alone). Alphabets indicate significant differences across groups at α<0.05. Superscript (a) indicates significant difference when compared to GroupA (Normal Feed alone), superscript (b) indicates significant difference when compared to GroupB (Cocoa Feed alone) while superscript (c) indicates significant difference when compared to GroupC Normal Feed + 8% salt + Uninephrectomy).

Cardiac and renal hydrogen peroxide levels were significantly elevated in high salt diet and uninephrectomized untreated rats when compared with the normal control and the cocoa powder alone. The uninephrectomized treated with cocoa powder and the uninephrectomized alone groups showed lower values of hydrogen peroxide levels when compared with the untreated group (Figure-1). Cardiac and renal MDA and PCO levels were significantly elevated in high salt diet and uninephrectomized untreated rats when compared with the normal control and the cocoa power alone. The cocoa treated and the uninephrectomized alone groups showed lower values of MDA and PCO levels when compared with the untreated group (Figures-2 and 3).

### Hemodynamic parameters

**Figure-4:**
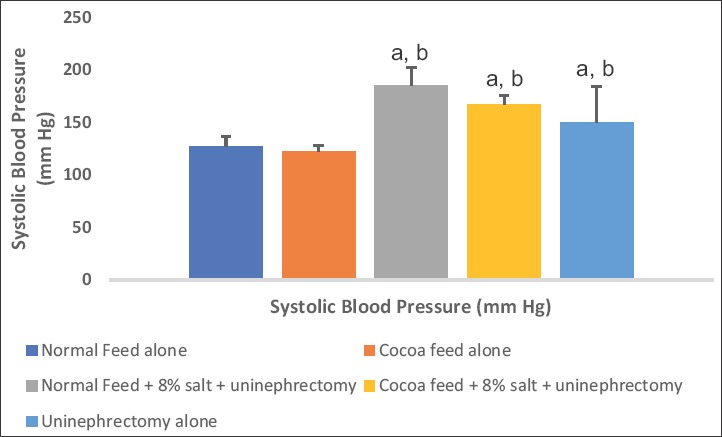
Effect of cocoa on systolic blood pressure (SBP). Values presented as mean ± S.D (n=10). GroupA (Normal Feed alone), GroupB (Cocoa Feed alone), GroupC Normal Feed + 8% salt + Uninephrectomy), GroupD (Cocoa Feed + 8% salt + Uninephrectomy), GroupE (Uninephrectomy alone). Alphabets indicate significant differences across groups at α<0.05. Superscript (a) indicates significant difference when compared to GroupA (Normal Feed alone), superscript (b) indicates significant difference when compared to GroupB (Cocoa Feed alone) while superscript (c) indicates significant difference when compared to GroupC Normal Feed + 8% salt + Uninephrectomy).

**Figure-5:**
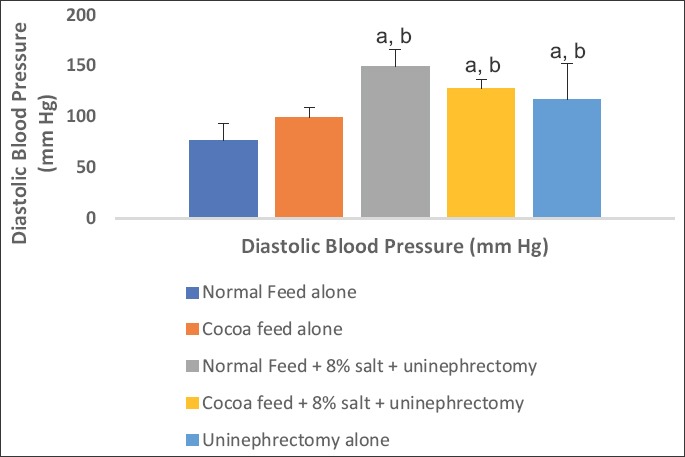
Effect of cocoa on Diastolic blood pressure (DBP). Values presented as mean ± S.D (n=10). GroupA (Normal Feed alone), GroupB (Cocoa Feed alone), GroupC Normal Feed + 8% salt + Uninephrectomy), GroupD (Cocoa Feed + 8% salt + Uninephrectomy), GroupE (Uninephrectomy alone). Alphabets indicate significant differences across groups at α<0.05. Superscript (a) indicates significant difference when compared to GroupA (Normal Feed alone), superscript (b) indicates significant difference when compared to GroupB (Cocoa Feed alone) while superscript (c) indicates significant difference when compared to GroupC Normal Feed + 8% salt + Uninephrectomy).

**Figure-6:**
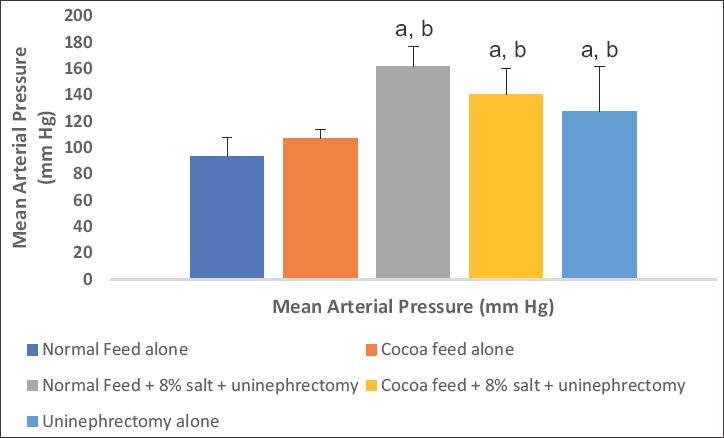
Effect of cocoa on mean arterial pressure (MAP). Values presented as mean ± S.D (n=10). GroupA (Normal Feed alone), GroupB (Cocoa Feed alone), GroupC Normal Feed + 8% salt + Uninephrectomy), GroupD (Cocoa Feed + 8% salt + Uninephrectomy), GroupE (Uninephrectomy alone). Alphabets indicate significant differences across groups at α<0.05. Superscript (a) indicates significant difference when compared to GroupA (Normal Feed alone), superscript (b) indicates significant difference when compared to GroupB (Cocoa Feed alone) while superscript (c) indicates significant difference when compared to GroupC Normal Feed + 8% salt + Uninephrectomy).

The values of SBP, DBP, and MAP were significantly elevated in the 8% salt + uninephrectomized untreated, uninephrectomized alone, and cocoa treated groups when compared with the normal control and the cocoa control groups. The cocoa treated and the uninephrectomized alone groups showed significantly lower values when compared with the untreated group (Figures-4-6).

### Histology

**Figure-7:**
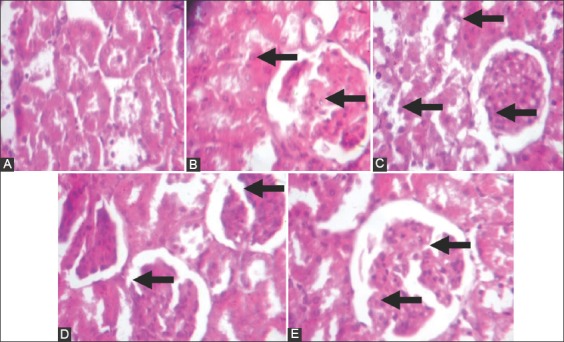
Histology of the Kidney: A Control (Normal rat feed): Plate shows mild glomerular congestion. B(Cocoa feed alone) Plate shows very mild focal congestion with moderate glomerular hypercellularity. C(Normal + 8% salt + Uninephrectomy) (Plate shows very mild focal congestion with moderate glomerular hypercellularity. D(Cocoa feed + 8% salt + Uninephrectomy) (Plate shows focal area of congestion and mild glomerular congestion. E(Uninephrectomy alone) (Plate shows mild glomerular congestion. Plates are stained with HandE stains and viewed with X 100 objectives.

**Figure-8:**
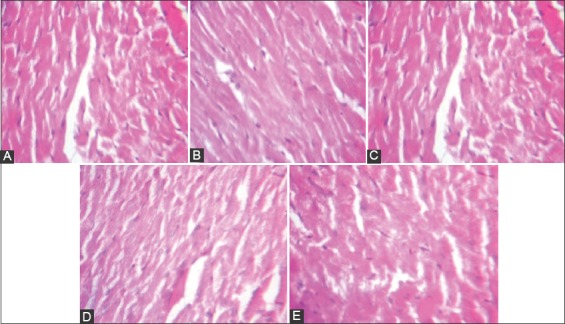
Histology of the heart: A Control (Normal rat feed): Plate shows no visible lesions. B(Cocoa feed alone): Plate shows no visible lesions. C(Normal + 8% salt + Uninephrectomy): Plate shows no visible lesions. D(Cocoa feed + 8% salt + Uninephrectomy): Plate shows no visible lesions. E(Uninephrectomy alone): Plate shows no visible lesions. Plates are stained with HandE stains and viewed with X 100 objectives.

Histology of the kidney showed mild glomerular congestion in the normal control group, and mild focal congestion with moderate glomerular hypercellularity in the cocoa control group, while very mild focal congestion with moderate glomerular hypercellularity in the 8% salt diet and uninephrectomized untreated group, focal area of congestion and mild glomerular congestion in the cocoa treated group and mild glomerular congestion in the uninephrectomized. Histology of the heart showed no visible lesions in all the groups (Figures-7 and 8).

## Discussion

The imbalance in the levels of antioxidants and reactive oxygen species (ROS) favoring ROS in the biological system termed oxidative stress has been incriminated in the pathogenesis and pathophysiology of various disorders of cardiovascular disease conditions and hypertension [38]. Reports have shown that oxidative stress is elevated in hypertension, and some antihypertensives such as β adrenergic blockers, calcium channel blockers, and ACE inhibitors are known to possess antioxidant properties, thus, asides from their primary mechanism of action, also target reduction in oxidative stress [39]. Sufficient evidence links oxidative stress in the kidney, heart, brain, and endothelium with hypertension, and interplay of the damage in these organs is thought to be involved in the development of hypertension [40]. The kidney is of particular importance in hypertension, and the direct connection between increased salt intake and high blood pressure has lent credence to the involvement of the kidneys in hypertension. [41]. Possible mechanisms through which oxidative stress induce hypertension include generation of vascular lipid peroxidation products, damaging endothelial and smooth muscle cells, and inflammation [42]. Our data showed increased levels of hydrogen peroxide generation, PCO contents, and lipid peroxidation products in the renal and cardiac PMF precipitated by uninephrectomy and high salt diet. The increases in hydrogen peroxide levels have been reported to activate NF-kB, and MAPKs pathways, which further complicate the oxidative injury. Stress-induced release of hydrogen peroxide has also been linked to Nrf2 activation[43]. Important defense systems such as SOD, CAT, and peroxidases were depleted in the 8% salt + uninephrectomy untreated group. Although these antioxidants play an essential role in maintaining and preventing the buildup of ROS, their counteracting effect is diminished in the presence of overwhelming pro-oxidants, and subsequent tissue damage follows[44].

Various research reports have pushed for a reduction of sodium chloride in various consumer products as it has been directly linked to the development of hypertension, cardiovascular, and renal diseases, while others advocated for the replacement of sodium with potassium and calcium-based salts[45]. The ability of the kidneys to excrete sodium chloride is directly related to the extracellular fluid and blood pressure. It takes longer for the kidneys to adjust to changes in dietary sodium chloride in compromised kidneys, as seen with uninephrectomy, old age than it does in normal kidneys. The extracellular fluid is elevated with an increase in sodium levels; this explains why a reduction in salt brings about a decrease in blood pressure[46]. From our findings, 8% salt aggravated blood pressure, markers of oxidative stress, and depleted the level of both enzymatic and non-enzymatic antioxidants. This finding corroborates other reports that have incriminated high salt diet in the pathogenesis of hypertension[47,48]. However, in the present study, uninephrectomy alone caused milder damage on the heart and kidney when compared with the damage caused by 8% salt and uninephrectomy. While this supports the earlier assertion that uninephrectomy is not linked to any major undesirable effect on the renal and cardiovascular system, it does not maximally reduce the quality of life, and that patients who have undergone uninephrectomy could live to full years[49]. It has been shown that uninephrectomized individuals are prone to developing salt-sensitive hypertension with consequent renal and cardiac damage. Uninephrectomy in our study must have increased salt-sensitive hypertension seen in the 8% salt + uninephrectomy untreated group. Although, uninephrectomy alone might have caused a mild increase in blood pressure and markers of oxidative stress when compared with the 8% salt and uninephrectomy untreated group, which must have exacerbated the effects in the untreated group. This assertion corroborates an earlier finding that uninephrectomy might give protection against an increase in blood pressure [1]. Aprevious study linked the blood pressure increases the effect of uninephrectomy to its interference with L-arginine metabolism[17]. L-arginine is a precursor of nitric oxide, which is known for its vasodilating effect. In our study, however, there was no significant difference in nitric oxide levels across the groups. This study, therefore, supports the earlier reports on the ability of cocoa to prevent endothelial dysfunction and thereby improving serum nitric oxide bioavailability [11,12].

Unless some form of intervention is given, hypertension almost always leads to heart failure, and the absence of hypertension lowers the risk of heart failure by 86%. The occurrence of renal and heart failure as a consequence of hypertension has thus led to the concept of cardiorenal syndrome [50]. The treatment for hypertension that would mitigate both the kidney and the heart complications is therefore necessary. In this study, we discovered that animals fed with cocoa significantly alleviated cardiac markers of oxidative stress and cardiac damage while improving cardiac enzymatic and non-enzymatic antioxidant defense system. MPO acts as a catalyst in the production of various ROS. Overwhelming evidence has connected MPO derived oxidants to the pathogenesis of cardiovascular diseases, making MPO a desirable target in therapeutic interventions involving the heart [51]. In our study, MPO levels were markedly increased in the 8% salt + uninephrectomy untreated group when compared with the other groups. Our results are in accordance with the previous findings of the anti-inflammatory properties of cocoa powder[9]. Apostulation of MPO’s mode of action is that it acts by catalyzing lipid peroxidation[51]. This is confirmed by the significant increase in cardiac and renal levels of MDA in the 8% salt + uninephrectomy, uninephrectomy alone groups. AOPPs are an indication of oxidative damage, occurring as a result of plasma protein oxidation and leading to renal and cardiac diseases [52]. The significant increase in serum levels of AOPP in the 8% salt + uninephrectomy untreated thus confirms that AOPPs can be used as a marker of cardiac damage. The present study, therefore, confirms the antioxidant property of cocoa due to the high polyphenol content of cocoa as previously reported [4].

PCO is the most universal and reliable pointer of protein damage and its stability under suitable conditions, and this makes it particularly useful as an indicator of organ damage [53]. The significant increase in its levels in the 8% salt + uninephrectomy untreated group when compared to the other groups suggests that high salt diet and uninephrectomy have damaging effects of protein structure and functions such as protein cross-linking and nitrosylation.

The high content of antioxidants in cocoa has made it a desirable compound in health management. It is known to contain quite a number of flavonoids, the most important one being epicatechin, which has therapeutic effects on myocardial infarction and ischemia by inhibiting NADPH oxidase, thus blocking the generation of ROS [54]. Its protective effect against DNA damage through its modulation of CYP450 by reducing oxidative stress and inflammation has been documented [55]. Its blood pressure-lowering ability has also been reported [56]. Acontrary report, however, said that cocoa has no effect on the blood pressure, weight, glucose, and lipid metabolism [57]. Interestingly, we observed that rats administered cocoa powder exhibited the lowest levels markers of oxidative stress, thus, justifying the decreased oxidative stress seen in the treated group. However, the exact mechanism of action of cocoa powder has not been fully explored; the high flavanol content of cocoa is thought to be responsible for its antihypertensive effect.

## Conclusion

Our findings lend credence to the involvement of renal and cardiac oxidative stress in high salt diet and uninephrectomy-induced hypertension and that cocoa powder mitigated the observable pathology. It also justifies the regular consumption of cocoa and its products as cocoa acts by ameliorating the generation of ROS in the renal and cardiac mitochondria. The findings of this study suggest an important role for cocoa in the management of hypertension and its complications.

## Authors’ Contributions

OCJ, AAO, OIO, TOO, TOA, OO, OOF, BSO, FBB, and OOF gave the concept, design, definition of intellectual content, clinical studies, experimental studies, data acquisition, data analysis, statistical analysis. ABS, AAA, OOO, MAY, and AO did manuscript editing and manuscript review.
